# Beneficial Autoimmunity at Body Surfaces – Immune Surveillance and Rapid Type 2 Immunity Regulate Tissue Homeostasis and Cancer

**DOI:** 10.3389/fimmu.2014.00347

**Published:** 2014-07-22

**Authors:** Tim Dalessandri, Jessica Strid

**Affiliations:** ^1^Division of Immunology and Inflammation, Department of Medicine, Imperial College London, London, UK

**Keywords:** immune surveillance, Type 2 immunity, epithelial cells, tissue homeostasis, carcinogenesis, IgE, intraepithelial lymphocytes, sterile stress

## Abstract

Epithelial cells (ECs) line body surface tissues and provide a physicochemical barrier to the external environment. Frequent microbial and non-microbial challenges such as those imposed by mechanical disruption, injury or exposure to noxious environmental substances including chemicals, carcinogens, ultraviolet-irradiation, or toxins cause activation of ECs with release of cytokines and chemokines as well as alterations in the expression of cell-surface ligands. Such display of epithelial stress is rapidly sensed by tissue-resident immunocytes, which can directly interact with self-moieties on ECs and initiate both local and systemic immune responses. ECs are thus key drivers of immune surveillance at body surface tissues. However, ECs have a propensity to drive type 2 immunity (rather than type 1) upon non-invasive challenge or stress – a type of immunity whose regulation and function still remain enigmatic. Here, we review the induction and possible role of type 2 immunity in epithelial tissues and propose that rapid immune surveillance and type 2 immunity are key regulators of tissue homeostasis and carcinogenesis.

Epithelial cells (ECs) are the main constituent of tissues lining body surfaces like the skin, intestine, lungs, and genitourinary tract. They regulate crucial life processes such as micronutrient absorption, gaseous exchange, and thermo- and hydro-control whilst also providing a physiochemical barrier to the external environment against microbes and a plethora of non-microbial stressors. ECs are extremely dynamic and versatile cells and it is becoming increasingly clear that they are also intimately involved in the induction and regulation of local tissue- and systemic immune responses. Disruption of epithelial surfaces may therefore result in dysregulated body processes and penetrance to deeper tissues by microbes or noxious moieties. In addition, the direct response of ECs to tissue disruption strongly affects resident immunocytes and their subsequent regulation of both local and systemic innate and adaptive immunity. A growing body of evidence both from mouse models and human genetics suggest that EC dysregulation can be a primary cause of pathology in different tissues. Given the multifaceted biological actions of ECs and the multitude of challenges imposed on epithelial tissues, it is reasonable to think that ECs in conjunction with tissue-resident immunocytes possess mechanisms, both immunological and non-immunological, to maintain healthy barrier homeostasis and to minimize inflammation and cellular dysregulation. Indeed, ECs are now known to be highly immunomodulatory by virtue of the cytokines, chemokines, damage-associated molecular pattern (DAMP) molecules, and major histocompatibility (MHC) gene products they express; a repertoire that has collectively been termed the “epimmunome” (1). ECs express pattern-recognition receptors (PRRs) including toll-like receptors (TLRs), NOD-like receptors (NLRs), RIG-I-like receptors (RLRs), and a variety of “NK” receptor ligands, enabling them to respond to a wide variety of microbial and non-microbial (including self) moieties and disseminate the response to immunocytes. The NLR–inflammasome complex allows ECs to respond to non-microbial sterile stress elicited by toxins, irritants, and (for skin keratinocytes) ultraviolet (UV) light (2); the most pervasive environmental DNA-damaging agent (3). Thus, ECs express a suite of sensors for detecting differing insults and challenges at the body surfaces, and an armory of soluble and cell-surface molecules to direct an appropriate, restorative response. These epithelial-driven responses in health sculpt and modulate tissue homeostasis and local tissue immunity, in a manner that aids morphological tissue homeostasis, restoration of the epithelial barrier following injury, and elimination or expulsion of microbial and non-microbial insults. Here, we review how ECs drive immunity at body surfaces and how this is involved in regulating immune surveillance, tissue immune homeostasis, and cancer.

## Epithelial Cells and Their Response to Challenge

The vast majority of environmental challenges occur at epithelial surfaces. The repertoire of responses available to ECs to combat these daily challenges is immense. For example, EC-derived cytokines include IL-1, IL-6, IL-10, IL-18, IL-25, IL-33, TNFα, and thymic stromal lymphopoietin (TSLP). Pro-IL-1α and pro-IL-1β are constitutively produced by ECs, particularly skin keratinocytes, and are secreted following exposure to noxious stimuli or tissue damage. Corneocytes (non-nucleated skin ECs) release IL-1α in the skin in response to disruption of the outermost surface, the stratum corneum (4), while UV-irradiation induces IL-1β. In addition to agonistic effects on tissue macrophages, IL-1α induces growth-factor expression from tissue fibroblasts, prompting a replicative burst in neighboring ECs to repair damage (5). The pro-inflammatory cytokines IL-6 and TNFα are produced in large quantities by damaged ECs; the former of which can also be used as a STAT-3-dependant autocrine growth factor, in healthy and cancerous epithelium (6). The most robustly expressed cytokines upon any EC insult however are IL-25, IL-33, and TSLP. In common, these three cytokines can drive type 2 immune responses, which thus impart a particular propensity of epithelial tissues to induce type 2 immunity. Such predisposition of stressed ECs may underlie the high frequency of allergic and atopic disease at the skin and mucosal surfaces. However, despite the intense interest in this area, the cellular and molecular linkage of type 2 immunity to barrier- and EC disruption is not clearly understood – nor is the functional role of this type of immunity to EC homeostasis or immune surveillance yet fully elucidated.

IL-25, also know as IL-17E, is a member of the IL-17 cytokine family. Despite bearing some amino acid sequence homology to the best-characterized IL-17 cytokines, IL-17A and IL-17F, IL-25 has divergent biological functions and promotes Th2 rather than Th17 responses *in vivo*. IL-25 directly amplifies expression of the Th-2 mediators IL-4, IL-5, and IL-13, and supports production of Th2 serum immunoglobulins (7). IL-25 was first reported with high steady-state mRNA expression in the kidney, and moderate to low expression in other organs and the peripheral tissue (8). Subsequently, it was found by multiple groups to be important in type 2-mediated immunity to enteric parasites, such as *Trichuris muris* (9), and is upregulated in the gut upon EC-sensing of commensal bacteria (10). The mouse gut parasite *Heligmosomoides polygyrus bakeri* elicits the EC-derived cytokine, IL-1β, which suppresses IL-25 and IL-33 and promotes pathogen chronicity by attenuating expulsive type 2 responses (11), suggesting that IL-25 is particularly important in maintaining immunity to gut pathogens. Similarly, mice and humans subjected to parenteral nutrition have impaired mucosal immunity, due to reduced gut luminal levels of antimicrobial effectors, but administration of exogenous IL-25 to parenteral nutrition-fed mice was found to be protective against enteric bacterial invasion (12). In allergic models, IL-25 expression is upregulated upon exposure to allergens both in murine or human lung EC lines and in primary murine lung ECs (13). Elevated protein levels have also been found in tissues of patients with allergic disease in the lung and skin (14). IL-25 has been found to drive tissue (airway) remodeling, and expression of the other major EC cytokines IL-33 and TSLP in a house dust mite model of allergy (15), and drive pulmonary fibrosis by inducing IL-13 expression from lung innate lymphoid cells (ILCs) in mice challenged with lung *Schistosoma mansoni* eggs (16). In addition to production by ECs, dermal dendritic cells (DCs) have been reported to be a major source of IL-25 in atopic dermatitis (AD) patients (17), while IL-25 and IL-33-activated ILC2s in mouse skin promote AD-like inflammation (18). These reports and others highlight an interesting crosstalk and autocrine regulation of EC-derived effectors, as well as a role for IL-25 in augmenting epithelial barrier immunity, or conversely promoting pathological Th2 tissue inflammation, in differing settings.

IL-33 is a multi-functional protein. The full length protein is localized in the nucleus but following cleavage the c-terminal fragment acts as a cytokine which binds the receptor ST2. IL-33 was recently discovered as an IL-1 family member with type 2-promoting functions similar to IL-25. It is expressed by ECs, macrophages, DCs, and mast cells *in vivo* and its cytokine function drives IL-4, IL-5, and IL-13 expression and differentiation of Th2 CD4^+^ T cells (19). IL-33-induced IL-4 production appears to be mainly from innate cells and together these two cytokines will induce proliferation of B cells and amplify IgE synthesis (20). Similar to IL-25, IL-33 acts in an autocrine fashion to promote TSLP expression by ECs, particularly in response to gut nematodes, where IL-33 mRNA can be detected rapidly following colonization (21). Interestingly, the efficacy of IL-33 in this infection model (and others) seems to be highly time-dependent, with administration of exogenous IL-33 at late time points post-infection being ineffective in promoting type 2 responses that would otherwise resolve infection. IL-33 is highly expressed by intestinal ECs and inflammatory infiltrates in ulcerative colitis, with IL-33 cleavage products being detected in the serum (22). IL-33 is also rapidly released and detectable in bronchoalveolar lavage fluid following lung allergen exposure in humans, suggesting it is a rapid type 2 mediator in sites additional to the gut (23). Protective as well as immunopathological roles of EC-derived IL-33 have been described in the skin. Transgenic over-expression of IL-33 in mouse skin, driven by a keratinocyte-specific promoter, induces a spontaneous dermatitis-like disease and activates ILC2s in the dermis (24). It has also been shown in a phorbol 12-myristate 13-acetate model of skin inflammation that mice deficient for the IL-33 receptor, ST2, do not exhibit IL-33-dependant skin inflammation (25). Similarly in human inflammatory conditions, IL-33 has been reported to be upregulated in clinical psoriatic lesions and the serum of skin sclerosis patients (26). Conversely, mice treated with exogenous IL-33, following full-thickness skin wounding, demonstrate dramatically improved wound-healing, collagen deposition, and expression of extracellular matrix proteins indicative of tissue repair (27). These reports suggest a particularly rapid and acute role for IL-33 in cutaneous homeostasis and gut integrity whereas constitutive, late, or dysregulated expression may be involved in a variety of chronic inflammatory conditions. This temporally coordinated aspect fits well with current thinking of IL-33 as an “alarmin,” whereby its immediate release from intranuclear stores by damaged, apoptotic, or necrotic cells rather than a classic Golgi-mediated secretion pathway (19) facilitates a rapid and restorative response to tissue damage.

TSLP is produced almost exclusively by ECs of the lung, tonsils, intestine, and skin (13), and is upregulated in response to tissue damage (28), various TLR ligands and infection, or exposure to type 2 cytokines such as IL-4, IL-13, IL25, and IL-33 (29). A protective role of TSLP in intestinal immunity to *T. muris* has been well described; mice which are knockouts for IKKβ fail to produce TSLP in response to infection, and subsequently develop chronic intestinal inflammation (30). Mechanistically, EC-derived TSLP suppresses p40 and upregulates OX40L expression in DCs, a costimulatory molecule with a propensity to license Th2 responses in CD4^+^ T cells (31). TSLP also augments Th2 cytokine production by direct effects on CD4^+^ T cells and has indirect, agonist effects on a variety of granulocyte populations including mast cells and basophils. Similar to IL-25 and IL-33, inappropriate expression or dysregulation of TSLP is implicated in a number of inflammatory diseases including the triad of atopic diseases; asthma, allergic rhinitis, and AD (31). TSLP is required for allergic lung inflammation in mice exposed to inhaled antigen, and TSLP receptor knockout animals do not develop lung inflammation in this model. Interestingly, these animals do develop strong Th1 responses with high IFNγ, IL-12, and IgG2a (32), highlighting how a single epithelial-derived molecule can skew adaptive immune responses in response to tissue-challenge. In humans, AD sufferers show high TSLP expression in lesional skin (33), and mice with induced expression of TSLP in the epidermis develop spontaneous AD-like pathology (34). Production of TSLP is however critically important for resistance to skin carcinogenesis in mouse models (35, 36).

Further to cytokines and chemokines, ECs can release other proteins upon cellular stress. Of note, they can produce hedgehog morphogens, which are a family of secreted proteins that regulate a wide variety of physiological processes including tissue development during embryogenesis and tissue homeostasis as well as being implicated in carcinogenesis (37). Sonic Hedgehog h (Hh) expression was recently found to be upregulated in lung ECs in models of allergic disease, and lung resident T cells were shown to respond locally to EC-derived Hh by upregulating IL-4 (38). This demonstrates that ECs also produce non-classical immune modulators, such as tissue morphogens, which appear to contribute to the robust induction of type 2 immunity in epithelial tissues.

In addition to the secreted and soluble molecules produced by ECs, they also express a variety of cell-surface molecules enabling them to directly interact with resident and infiltrating immunocytes. For example, ECs express E-cadherin that engage CD103, which is constitutively expressed on intraepithelial lymphocytes (IELs) and tissue DCs such as the epidermal Langerhans cells (LCs). ECs also express T cell costimulatory ligands, although it remains unclear as to what extent ECs express the classical B7.1 and 2 molecules, they clearly express PD-L1 and PD-L2 (39). Some members of a novel family of B7-related molecules, the butyrophilins, appear to be preferentially expressed on ECs and have been implicated in EC-immune regulation (40) as have *Skint* family members which are exclusively expressed on ECs and have profound impact on IEL development and function (41, 42). Thus via appropriate receptor–ligand interactions ECs are capable of initiating and sculpting both local tissue immunity and further downstream systemic immunity. Under conditions of physiochemical tissue disruption or barrier perturbation (1), infection (43), genotoxic stress (44), sterile inflammation, or heavy proliferation (45), ECs respond by upregulating additional self-encoded and cell-surface markers, which are often termed as “‘stress antigens” as they are indicative of a dysregulated state of the epithelium. The EC stress antigens have an important role in initiating and directing tissue immune responses during perturbations and as such these will be discussed in more detail in the Section “Immune Surveillance” below.

## Epithelial Cells and Their Neighbors

In close association with ECs, the epithelial tissues are home to several specialized subsets of immunocytes. IELs are found in all epithelial tissues, but have most notably been studied in the intestine and skin. IELs are adaptive T cells carrying RAG-dependent rearranged T cell receptors (TCRs), nevertheless they are often MHC non-restricted cells and express many innate receptors allowing them to react to stress antigen with “innate-like” response kinetics. The IELs are a mixture of αβ and γδ T cells, which are either CD4^−^CD8^−^ or coexpress a CD8αα coreceptor. The ratio of αβ to γδ T cells depends on the anatomical site as well as the species. IEL compartments are often much less diverse than systemic T cells (for example in the mouse skin and uterus they are essentially monoclonal), implying that these cells recognize predictable antigens encountered in specific tissues – these antigens could be either pathogen encoded or self-encoded molecules that reflect a dysregulated state of the tissue they inhabit. Not many IEL TCR-specificities have yet been defined, but it seems clear that both their recognition capabilities and mode of activation are distinct from systemic T cells (46). Particularly, it has been proposed that IELs are primarily autoreactive T cells that have been agonist-selected, recognize tissue stress antigens, and have regulatory properties (47). The murine skin for example contains a specialized subset of γδ (TCR)^+^ IELs called dendritic epidermal T cells (DETC) that exclusively carry a Vγ5Vδ1 TCR – a TCR arrangement only found on epidermal IELs (and on the progenitor fetal thymic population). The skin epithelia also contain a specialized subset of DCs, the epidermal LCs. Both LC and DETC infiltrate the epithelium very early during stratification of the skin ECs, and are long-lived and likely self-renewing immune compartments, which clearly integrate and physically interact with the ECs.

In addition to the “unconventional” T cells in the epithelium, more conventional CD8αβ^+^ αβ T cells have been shown to rapidly accumulate in tissues upon infection, where they can become resident memory T cells and provide protective antigen-specific responses. This was elegantly shown in the skin following local infection with herpes simplex virus (48) or vaccinia virus (49), which induced a rapid influx of antigen-specific CD8^+^ αβ T cells both into the epithelial epidermal layer and the underlying dermis. Interestingly, these infiltrating CD8^+^ αβ T cells were shown to populate the entire skin and provide long-lasting protection against re-infection as a continuing tissue-resident memory T cell population. In steady state, the subepithelial layer of most tissues contains a diverse set of immunocytes that can all contribute to epithelial-immune surveillance. These include tissue-specific resident populations of myeloid cells, such as DC, macrophages and mast cells, lymphoid cells, such as CD4^+^ or CD8^+^ αβ T cells, γδ T cells, and ILCs, as well as stromal fibroblasts. In fact, it is becoming increasingly apparent that different tissues constitutively harbor a variety of specialized immunocytes in the subepithelial space. For example, in human skin a population of IL-22 and growth factor producing T cells (Th22) can rapidly enter the epithelium upon challenge and be involved in epidermal remodeling (50). Similarly, the gut contains a resident IL-22 producing population of NK-like cells (51). In recent years, an array of different ILCs has been discovered that are resident in the subepithelial tissue layer. Different subsets of ILCs dominate in particular tissues and their specialized functions are starting to be elucidated; many of them contribute to both homeostatic and pathophysiological conditions in the tissue they inhabit (52). The subepithelial immunocytes can respond to epithelial cues and be recruited into the epithelium upon damage – in addition systemic immune cells can be recruited both to the subepithelial and epithelial layer. In sum, the epithelium and body surface tissues are home to an intricate array of immunocytes, which can interact and integrate activities in numerous complex ways that likely differ substantially depending on the anatomical site and the challenge encountered. Additional research is required to understand the interaction between different resident immunocytes in the tissues and how their responses may be integrated to regulate local and systemic immunity.

## Type 2 Immunity and Its Triggers

**Figure 1:**
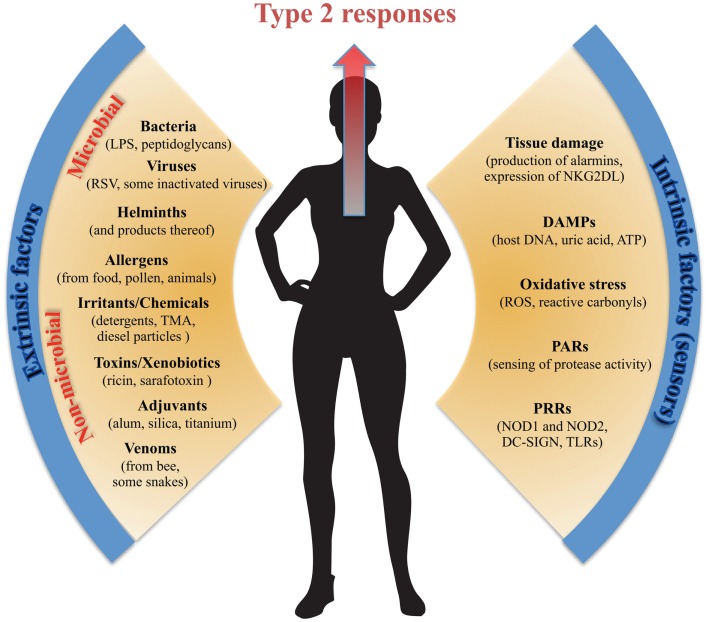
**Extrinsic and intrinsic factors promoting type 2 immunity**. Type 2 immunity can be triggered by an array of diverse extrinsic stimuli from both infectious and non-infectious sources and is most potently induced at the body surface tissues. Equally, the intrinsic cellular mechanisms inducing and/or sensing type 2-triggering extrinsic stimuli are many and diverse. In common, most of the extrinsic factors promoting type 2 immunity, as well as the intrinsic factors sensing them, are founded on a breach of the protective barrier of the body and thus on tissue and cellular damage.

Epithelial cells and EC-associated leukocytes such as IELs can clearly drive local and systemic type 2 immunity (53). More conventionally, however, type 2 immunity is thought to be mediated primarily by Th2 cells, IgE and IgG1 antibodies, as well as a host of innate immune cells such as mast cells, basophils, eosinophils, alternatively activated macrophages, and ILCs. The type 2 immune response *in vivo* is accordingly extremely heterogeneous and it is surprisingly poorly understood how type 2 immunity is induced, regulated – and indeed what its primary physiological function is. Type 2 immune responses are classically induced by macroparasites and conventional thinking holds that type 2 immunity has evolved to protect against infection by parasites such as helminthes and ticks. However, this is probably a too simple explanation, as it is not true that all parasites are fought by IgE and type 2 immunity. Although IgE levels are raised in people as well as mice with helminth infections, IgE is dispensable for immunity to many helminthes and much of the IgE raised is not specific to the parasite (54). Type 2 immunity is also notoriously activated in response to a broad range of different environmental challenges and antigens. Such non-infectious stimuli that trigger type 2 immunity are collectively termed allergens and form the basis of a host of allergic disorders like asthma, allergic rhinitis, food allergies, and AD. Type 2 immune responses have been explored largely in the context of helminth infections and allergic diseases. They have been thought to provide a host-beneficial role only as defense against macroparasites, whereas allergic reactions are most commonly explained as a detrimental consequence of a misdirected response mimicking parasite immunity. This paradigm is now changing and with more triggers of type 2 immunity being elucidated (Figure 1) it seems plausible that type 2 immunity can provide host-benefits in settings other than against parasites. In 1991, Profet published an inspired hypothesis suggesting that the physiological role of allergic responses was an immunological defense against toxins (55). This idea is resonating with recent data (56, 57) and the hypothesis has been recharged and expanded into a broader model of intentional allergic host defense not only against helminthes but also non-infectious environmental factors such as venoms, chemical irritants, and xenobiotics (58). Accordingly, there may be multiple pathways that lead to type 2 immunity and IgE – some more classically “adaptive” and some more “innate” (59). The route to type 2 immunity and whether protection or allergic sensitization is the outcome may depend on tissue context, allergen, dose, genetics, and species. Common for all type 2 immune responses is that their effector functions converge at the epithelial surfaces (skin and mucosa), vasculature, and smooth muscles where they promote barrier defenses and expulsion. Conspicuously, allergic disorders, unlike other immune pathologies, exclusively affect epithelial tissues that interface with the environment.

Type 2 immunity can be triggered by a bewildering array of molecules from both infectious and non-infectious sources (Figure 1). Much work has been done to try and identify a unifying framework for what makes a substance “an allergen” (60), but common allergens such as peanut, shellfish, pollen, nickel, bee venom, latex, house dust mite, and penicillin appear to have little in common in terms of their chemical structure or origin. In addition, type 2 immunity can be triggered by certain vaccine adjuvants [alum most notably (61)], noxious toxins (56, 58), environmental irritants and chemicals (62, 63), as well as certain infections or bacterial products (64). One commonality between both infectious and non-infectious triggers of type 2 immunity that may have been less well appreciated is that many are insults inducing some level of physical trauma that breaches the protective barrier of the body. Tissue damage, at least in the absence of strong type 1-promoting pathogen-associated molecular pattern (PAMP) signaling, appears to be a potent mechanism driving type 2 immunity. Tissue damage induces rapid release of several epithelium-derived cytokine alarmins, such as IL-33, TSLP, and IL-25 (reviewed above) – all of which can drive downstream type 2 immunity. In a macroparasite infection, the large size of the parasite and the consequent tissue damage it causes during invasion may be the most important factor in inducing type 2 immunity (65), although some parasite-derived products with direct type 2 polarizing capacity may also exist (66). The tissue-damage caused by macroparasites may be modeled by ingestion of large inert particle structures. Interestingly, it has been shown that inert silica and titanium particles induce innate type 2 immunity and can be used as adjuvants promoting Th2 responses by pathways independent of TLR4 and MyD88 (67, 68). These particles may induce cellular damage and consequently activate endogenous danger- or stress-signals. That “injury” or cellular stress alone can support induction of type 2 immunity is strongly supported by results showing that transgenic up-regulation of the NKG2D stress-ligand Rae-1 on ECs promote potent type 2 immunity and IgE to innocuous antigens (53). This rapid innate-like IgE response is also independent of MyD88 (53). Cellular damage may also explain the type 2-inducing effect of the adjuvant alum as injection of alum causes the release of DAMPs, like uric acid and host cell DNA (61, 69). Uric acid has been shown to drive type 2 immunity and again this is via pathways independent of both MyD88 and the inflammasome (69). Host DNA signaling intriguingly appears to differentially regulate IgG1 and IgE production following alum-adjuvanted immunization, where host DNA induces primary B cell responses with IgG1 through interferon response factor 3 (Irf3)-independent mechanism but more canonical Th2 responses and IgE through an Irf3-dependent mechanism (70). Furthermore, extracellular ATP, presumably released from damaged cells, binds to P2 purinergic receptors and triggers IL-33 release and innate type 2 immune responses in the lung (71). Oxidative stress, which is widespread and entwined with pathological processes, has also been shown to be involved in orchestrating type 2 immunity. For example, induction of reactive oxygen species (ROS) in ECs induces oxidation of lipids that in turn triggers TSLP release by ECs (72) and oxidative stress has been shown to induce reactive carbonyl adduction, which is reported to be a potent driver of type 2 immunity (73). DAMPs thus appear to be part of both the initiation and amplification of type 2 immunity and may as such also play an important role in allergic diseases.

Another feature that contributes to the induction of type 2 immunity by some allergens is their serine or cysteine protease activity. Allergens such as Der P1 (from house dust mite) and papain (from papaya fruit) appear to rely on their protolytic function as inactive forms of these proteins do not induce type 2 immunity (60). A cysteine protease from the parasite *Leishmania Mexicana* has also been shown to induce type 2 immunity and this could be blocked by protease inhibitors (66). The importance of controlling enzymatic activity at epithelial surfaces is dramatically demonstrated in patients with Netherton syndrome. Netherton syndrome, which is caused by hereditary mutations in the serine protease inhibitor, LEKTI, presents with severe disruption in barrier function and persistent atopy, allergic disease, and AD (74, 75). The mutation in LEKTI results in persistent activation of protease-activated receptor (PAR)-2 and induction of TSLP and type 2 immunity (76).

Other endogenous stress-signals, for example the NLR receptors, NOD-1, and NOD-2, can polarize antigen-specific immune responses toward Th2 and thus contribute to the onset of adaptive immunity (77, 78). Interestingly, NOD-1 and NOD-2 expression within the stromal compartment is necessary to prime effector CD4^+^ Th2 responses and full Th2 induction is dependent on stromal TSLP (79). The type 2-inducing innate immune sensing is in these cases recognition of bacterial-derived products (peptidoglycans) and not self- or environmental antigens. Although the role of PAMPs and PRRs such as TLRs are usually associated with type 1 immunity, there are other examples in which TLR stimuli can induce type 2 responses. For example, low doses of lipopolysaccharides (LPS) have been proposed to promote Th2 cell responses (whereas high doses promote Th1) (80) for which stromal expression of TLR4 is critical (81). Certain microbial stimuli that signal via DC-SIGN induce Th2 biased responses and many TLR2 agonists have also been shown to suppress Th1 and promote Th2 responses (82). Furthermore, in the case of allergens, there is evidence that some can be directly sensed by PRRs; house dust mite allergens (83) as well as nickel (84) can signal via TLR4 for instance.

Given the vast array of molecules that can trigger type 2 immunity and the many innate and adaptive immune cells involved in orchestrating the response it seems reasonable that there are several routes to inducing type 2 responses and that these may yield a qualitatively different kind of type 2 immunity. The conventional mode of inducing type 2 immunity and high affinity antigen-specific IgG1 and IgE antibody has since long been described and substantiated. Activated CD4^+^ αβ Th cells upregulate CD40L and secrete IL-4 and IL-13, whereby they promote germ-line transcription of the γ1 and ε heavy chain to initialize class switching. This requires cognate interactions between B cell MHC II molecules and the TCR–CD3 complex. However, perhaps especially with regards to IgE, there appears to be additional non-conventional modes of inducing class switching and the requirement for T cell help may differ. In contrast to orthodox belief, mice that are deficient in αβ T cells have highly elevated levels of IgE antibodies and class switch particularly efficient to IgG1 and IgE (85, 86). Mice lacking the linker for activation of T cells (LAT) adapter protein (87) or the Tec kinase ltk also have elevated levels of IgE (88, 89), which may be regulated non-conventionally by γδ T cells. Evidence for a non-conventional route to IgE has also been demonstrated during the γδ T cell dependent “lymphoid stress-surveillance response” in the context of stressed skin epithelium (53). It has been established that the IgE produced in immunodeficient mice differ from conventional adaptive IgE not only by being MHC II-mediated T cell cognate independent but also by lacking dependence on germinal centers and thus producing IgE without significant somatic hypermutations (90). Moreover, this “natural” IgE also appears to be mainly self-reactive (85, 90). It may be that in a given circumstance a mixture of conventional adaptive routes and less-adaptive non-conventional routes to IgE are operating simultaneously. For example, infection with a helminth produces not only high affinity antigen-specific IgE but also a lot of “non-specific” IgE and similarly NKG2D-dependent induction of IgE from stressed skin produces not only antigen-specific IgE to an antigen encountered simultaneously but also “non-specific” IgE (53). Analysis of IgE repertoires and the particular requirements for development of IgE-secreting B cells is needed to further elucidate conventional (via Th2) and non-conventional routes to IgE. This may also provide invaluable information as to what actually constitutes a host-protective response (against tissue stress, toxins, parasites) versus allergic Th2 immunity.

## Immune Surveillance

To address the role of EC-driven type 2 immunity in tissue immune surveillance it is useful to first define “immune surveillance.” Immune surveillance refers to the capacity of the immune system to sense cellular dysregulation and respond by activating a stress response to restore homeostasis. This continued “quality control” mechanism has most commonly been applied to and studied in relation to cancer. The cancer immuosurveillance hypothesis was first proposed by Ehrlich in 1909 when he predicted that the immune system could repress or destroy the outgrowth of tumors that arise spontaneously on a continued basis (91). This proposal initiated a century of debate over the immune systems role in controlling neoplasia. The idea of a natural immune response against neoplasms or pre-malignant and dysregulated cells was revisited and expanded by Burnet and Thomas in the 1950s (92, 93). They proposed that lymphocytes form the basis of a “cancer immunosurveillance” process that protects immunocompetent hosts against primary tumor development. Although the hypothesis grew in recognition with the expansion of knowledge about the immune system and tumor-antigen recognition, the architects themselves pointed to “the problem with the idea of immunosurveillance is that it cannot be shown to exist in experimental animals” (94) – and it is of course rarely appreciated in a clinical setting. By the early 1990s, little attention was paid to the idea that natural immunity could control tumor establishment *de novo*. However, by the mid-1990s and onward several observations were made that rekindled the interest in this early aspect of tumor immunity [reviewed in Ref. (95)]. In short, the physiological importance of immune surveillance was well revealed by the pathological consequences of its failure: the neutralization of IFNγ with antibodies (96) and later the use of mice lacking IFNγ responsiveness was shown to enhance tumor growth (97). Lymphocytes were unequivocally proven to play an essential role in immune surveillance by seminal observations in *rag2*^−^*^/^*^−^mice (98) and subsets of lymphocytes such as NK, NKT cells (99), and γδT cells (100) were shown to play prominent roles in the control of malignancy. These new data prompted a refinement of the cancer immunosurveillance concept (95) and an ongoing quest to understand the triggers and mechanistic action of this early and continuous immune response against altered self.

## Elicitors and Effectors of Immune Surveillance

Epithelial-derived cancers, called carcinomas, make up about 85% of all cancers. The epithelial barriers of our body surfaces are also where the majority of exogenous stresses and challenges occur. Both sterile and microbial insults are encountered daily at epithelial surfaces and prompt EC and immune activation. Cancer development is, however, a multifactorial and multistep process. Most solid cancers only emerge following a sequential accumulation of somatic mutations over many years, which eventually may overwhelm the barriers that normally restrain their growth and thus clonal expansion of transformed cells can occur. Cumulative mutational load, telomere dysfunction, and altered stromal milieu are all required before a solid tumor presents (101). Fortunately, numerous intrinsic and extrinsic tumor-supressor mechanisms exist to prevent the development and outgrowth of malignant cells and all cells continuously undergo these rigorous “health checks.” The normal health control mechanisms can be triggered both by endogenous and exogenous stress and are executed by a cell-autonomous intrinsic surveillance system (such as delay in cell-cycle progression, repair of DNA-damage/genetic mutations, and induction of senescence or apoptosis) – and backed up by extrinsic immune surveillance mechanisms triggered by manifestations of EC dysregulation. The cell intrinsic responses to stress and the cell-extrinsic responses of the immune system are therefore intimately linked.

Damage-associated molecular patterns are mainly intracellular components of cells that are released or exposed upon physical or metabolic stress or cell death (102). For example ATP released from dying cells can act as a chemoattractant on macrophages drawing them to the stressed tissue (103). Extracellular ATP can bind to P2 purinergic receptors, which dependent on the cell engaged, can induce inflammatory (104) or anti-inflammatory (71) immune responses. Release of ROS or DNA from damaged cells can also powerfully initiate immune surveillance responses (105). Upon stress, ECs also rapidly and potently increase their synthesis of complement C3 (106), which due to its action on a multitude of innate and adaptive immune cells is likely to play a role in early immune surveillance, although its role in cancer as well as tissue homeostasis is as yet relatively unexplored.

In addition to the release of DAMPs, complement and cytokines/chemokines ECs can in response to numerous forms of cell-dysregulation dynamically alter cell-surface antigens to engage with receptors on innate and adaptive immune cells. Ligand–receptor interactions between ECs and tissue-resident immunocytes are thus important not only for homestatic interactions but are key regulators and elicitors of immune surveillance. One of the most important and best-characterized families of stress-induced EC ligands includes Rae-1, H60, and MULT1 (mouse), MICA, MICB and ULBPs (human). These are members of the larger family of MHC class Ib molecules and are reported upregulated on ECs by stresses such as heat-shock, UV-irradiation, DNA-damage, viral and bacterial infection, and autoimmunity. These unconventional MHC molecules engage the activating lectin-type receptor NKG2D, which is constitutively expressed by tissue-resident T cells and NK, NKT cells but is also expressed on CD8^+^ T cells and in some circumstances subsets of CD4^+^ T cells. The NKG2D-pathway has proven important in numerous settings of cell-dysregulation, such as cancer (107), infection (108), autoimmunity (109), and transplantation [reviewed in Ref. (110)], and its key role in immune surveillance is supported by the plethora of strategies tumors and viruses have adopted to evade it (111, 112). In relation to cancer, NKG2D-ligands are expressed by most epithelial tumors and the NKG2D-pathway is strongly associated with anti-tumor responses in both humans and mice (113). NKG2D-ligands are often upregulated early upon cellular dysregulation or transformation, it has however been controversial whether immune cells could be activated by such self-moieties alone. By generating transgenic mice where an autologous NKG2D-ligand, Rae-1, could be upregulated on keratinocytes by administration of doxycycline it was shown that even in the absence of any overt microbial stress (or overt tissue/cellular dysregulation as in a tumor setting) engagement of NKG2D on the epidermal IELs (DETCs) activated these cells and caused profound changes in the local immune compartment (114). This demonstrates that resident immunocytes can recognize and act solely on alterations in autologous stress antigens and thus survey the “health-status” of a given EC, pre-malignancy. The data support the cancer immune surveillance theory as it was also shown that the tissue-resident IELs have a key role in host-protection against skin carcinogenesis (114). Afferent sensing is normally attributed to innate myeloid cells, perhaps particularly to DCs that are often viewed as the primary orchestrator of adaptive immunity. To highlight the capacity of tissue-resident T cells (as demonstrated by the epidermal γδT cells discussed above) to perform an equivalent function as sensors of dysregulation, this mode of afferent sensing has been termed “lymphoid stress-surveillance” (115, 116). Lymphoid stress-surveillance may particularly be engaged in recognition of “stressed-self” and as such confer “beneficial autoimmune” responses in our body surface tissues. It is intriguing that NKG2D is expressed primarily, perhaps exclusively, by lymphoid cells (γδT, NKT, CD8^+^ αβT, and NK cells), suggesting that engagement of NKG2D could elicit an acute lymphocyte stress response to EC damage perhaps engaging different cells in different tissues.

In addition to the NKG2D-pathway, many other ligand–receptor pathways modulating epithelial-immune cell interactions and contributing to immune surveillance and tissue homeostasis are emerging. One such emerging family of regulators is the nectin and nectin-like (necl) proteins. Nectins are immunoglobulin-like cell–cell adhesion molecules involved in the formation of adherens junctions in ECs and fibroblast. Both nectin and the necl molecules play important roles not only in adhesion but also in migration, proliferation, and wound healing (117, 118). The group of receptors that engage these nectin molecules are therefore now being intensely studied in relation to cancer and immune surveillance (119). The major receptors that bind nectin and necl family members are DNAM-1 (CD226, PTA-1, TLiSA1), class I-restricted T cell-associated molecule (CRTAM), CD96, and TIGIT (WUCAM, VSIG9, Vstm3). All of these receptors are expressed on NK cells, γδT cells and CD8^+^ αβT cells and can mediate effector functions in these cells upon engagement. DNAM-1 ligands are frequently upregulated on tumor cells and have been reported to be regulated through the DNA-damage response pathway (120). Activation of DNAM-1 can evoke potent cytotoxicity in both T cells and NK cells (121) and control tumor growth (122). CRTAM binds necl2, which have been shown to regulate wound healing in the skin (118) and be involved in metastasis of human tumors (123). Expression and activation of CRTAM on immunocytes is likely highly important in early control of tissue homeostasis and cancer immune surveillance. *In vitro* studies have shown CRTAM to induce IFNγ from T cells and *in vivo* ncl2 expressing tumors have been shown to be controlled by NK and CD8^+^ T cells. Less is currently known about CD96 and TIGIT, but interestingly TIGIT appears to have an inhibitory function on NK and T cells (119).

Another example of an immune surveillance stimulator displayed by stressed ECs is the Coxsackie and adenovirus receptor (CAR). CAR is also a junctional adhesion molecule, it is upregulated on damaged ECs and potentially revealed when integrity of the tight junction is compromised. It binds junction adhesion molecule-like (JAML), which is expressed on neutrophils, tissue-resident γδT cells, and to a lesser extent on monocytes and some activated CD8^+^ αβT cells. Resident skin and intestinal γδT cells upregulate their expression of JAML upon tissue injury and binding of JAML to CAR lead to proliferation, cytokine, and growth factor production (124). Inhibiting costimulation of resident γδT cells by blocking JAML significantly delayed wound healing, akin to the total absence of these resident T cells, suggesting that CAR-JAML interactions are important for initiation of immune surveillance and tissue homeostasis. Interestingly, it has been shown that interaction of JAML with CAR recruits the central cell signal transducer PI3K, as is known for the αβT cell costimulator CD28, further emphasizing JAMLs role as a costimulator for tissue-resident T cells with implications for immune surveillance of dysregulated ECs (125).

Similar to the role of CAR-JAML interactions between ECs and resident T cells, it has recently been shown that plexin-B2-CD100 interactions are important for regulating the activity of IELs in both the skin and intestine (126, 127). CD100 (also know as Sema4D) is a member of the large family of semaphorin proteins. These proteins interact with plexins, which were first shown to play a fundamental role in the nervous system directing axon guidance. Intriguingly though, semaphorin–plexin interactions are also extensively involved in regulating immune responses and analysis of CD100-deficient animals have revealed a crucial role for this semaphorin in both humoral and cellular immunity (128). In relation to EC-immunocyte interactions, plexin-B2 is expressed on ECs in the epidermis and in the colon and interaction with CD100 on resident γδ IELs promotes wound repair in the skin (126) and protects against dextran sulfate sodium (DSS)-induced colitis in the intestine (127). In both tissues, CD100^−/−^ mice failed to mount a proliferative EC response to tissue damage, which was attributable to the lack of activation and growth factor production by the γδ IELs required to heal the epithelium.

Tissue-resident immunocytes are in a unique position to carry out a continued maintenance function such as tissue stress-surveillance. Innate immune cells have the capacity to recognize antigens that are displayed in tissues following a variety of stressors and can respond rapidly in large numbers without requiring clonal expansion. The early stages of an immune response – the afferent phase – are therefore conventionally ascribed to myeloid cells or NK cells. However, as highlighted above tissue-resident T cells can also be afferent sensors of cellular dysregulation. The importance of a tissue-specific resident population in cancer immune surveillance has nevertheless been difficult to verify. This was addressed experimentally by taking advantage of the unique tissue location of specific γδTCR-expressing IELs in the mouse, where the epidermal population of Vγ5Vδ1^+^ IELs can be specifically knocked out (leaving all other T cell populations intact). These *vg5vd1*^−^*^/^*^−^ mice are significantly more susceptible to cutaneous carcinogenesis than wild-type mice, demonstrating a key role for resident tissue-specific IELs in cancer immune surveillance. Consistent with the cancer immune surveillance hypothesis the γδ IEL act early and significantly suppress the development of papillomas but cannot suppress the progression from papilloma to carcinoma (114). Thus, myeloid cells, NK cells, and IELs all act as afferent sensors of dysregulation and initiators of immune surveillance in epithelial tissues. These cells can then also contribute to the downstream effector and regulatory phases of immunity. Clearly, both CD4^+^ and CD8^+^ αβT cells as well as B cells play important roles in cancer immune surveillance in the effector phase.

## Functions and Mechanisms of Immune Surveillance

The afferent phase of immune surveillance – the sensing of dysregulated self – applies to many other stresses than purely oncogenic stress. Thus the concept of immune surveillance not only pertains to cancer; accumulating evidence suggests that it can be more broadly applied to other non-malignant pathologies. For example, liver fibrosis, as a result of liver damage, is exacerbated when NK or NKT cells are depleted or the gene for perforin (required for cytotoxicity) is deleted as the stressed hepatic stellar cells cannot be controlled (129, 130). Stressed hepatic stellar cells express NKG2D-ligands upon damage, as described for ECs, facilitating their recognition by immune surveillance cells. Interestingly, natural activation of hepatic iNKT cells inhibits fibrosis whereas non-natural “over-stimulation” of iNKT cells appears to have the opposite effect and accelerate liver injury (129). Equally in the liver, tissue-resident macrophages have been shown to protect against ischemia reperfusion injury and be critical for tissue homeostasis (131).

Mice lacking normal resident IEL repertoires, such as *Tcrd*^−^*^/^*^−^mice, develop spontaneous chronic dermatitis, which can only be downregulated when *Tcrd*^−^*^/^*^−^ mice are reconstituted with the tissue-specific resident IEL (the Vγ5Vδ1 TCR-expressing epidermal DETC) (132). Interestingly, these *Tcrd*^−^*^/^*^−^mice also show a defect in the integrity of the epidermal barrier, as measured by hydration status and transepidermal water loss (TEWL). However, the epidermal barrier defect is obvious only upon environmental challenge, consistent with the notion that the IELs survey the health-status of the ECs and promote tissue homeostasis (133). Skin IELs were also the first T cells to be implicated in promoting EC growth. Closure of full-thickness skin wounds is significantly delayed in *Tcrd*^−^*^/^*^−^ mice, and this is attributed to the skin IELs capacity to rapidly produce EC growth factors such as insulin-like growth factor-1 (IGF-1) and keratinocyte growth factors (KGF) (134, 135). Intriguingly, it has also been shown in humans that T cells isolated from healthy or acutely wounded skin actively produce EC growth factors and participate in wound repair, whereas T cells from patients with chronic wound-healing problems are anergic and unable to produce EC growth factors (136). Similarly, *Tcrd*^−^*^/^*^−^mice lacking the γδ IEL population, which represents a major intestinal T cell population, are more susceptible to DSS-induced mucosal injury of the gut and demonstrate delayed tissue repair due to the lack of localized delivery of EC growth factors from the missing γδ IEL compartment (137).

The examples above clearly demonstrate that immune surveillance is not only a mechanism to control the development and outgrowth of tumors but is also a key regulator of tissue homeostasis more generally [reviewed in Ref. (138)]. The implication of immune surveillance mechanisms in the maintenance and re-establishment of tissue homeostasis thus broadens its scope and it is likely that similar cell-extrinsic immune surveillance mechanisms are important at disease-initiating (pre-disease) stages in many pathophysiological settings other than cancer.

**Figure 2:**
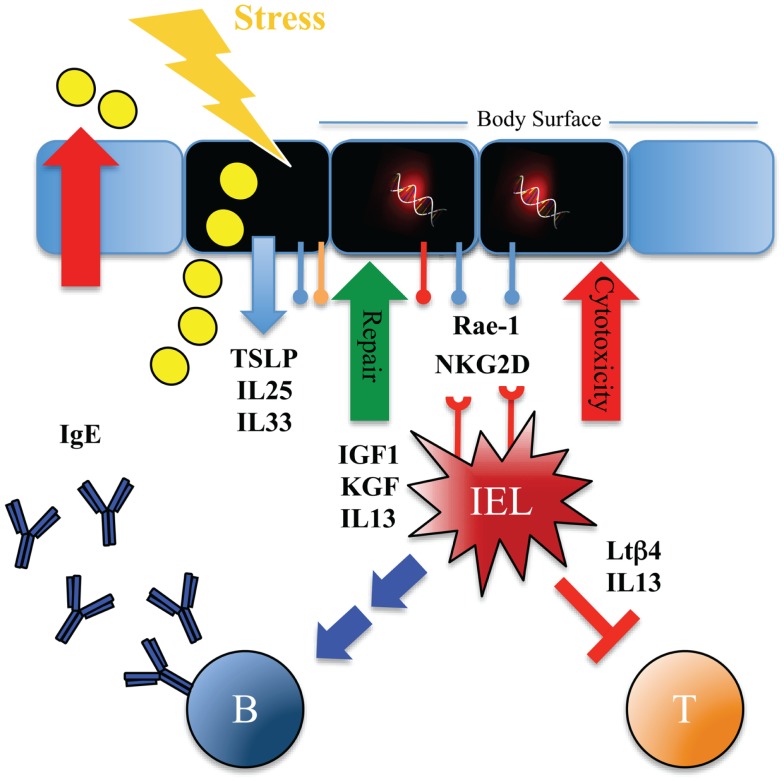
**Immune surveillance with type 2 immunity promotes tissue homeostasis and protects against carcinogenesis using numerous layers of control**. Scheme illustrating possible mechanisms whereby tissue-resident IELs can provide rapid host-protective immune surveillance and re-establish tissue homeostasis at body surfaces. Tissue stress as imposed by mechanical disruption, injury, or exposure to noxious environmental substances such as chemicals, carcinogens, UV-irradiation or toxins prompts ECs to release IL-25, IL-33, and TSLP cytokines, and upregulate expression of stress-ligands such as Rae-1, CAR, and plexins. This activates the resident IELs and their responses include cytolytic effects, release of growth factors (for example IGF-1, KGF), dampening of αβ T cell-mediated inflammation [for example by release of thymosin-β4 lymphoid splice variant (LTβ4)], rapid and potent production of IL-13, as well as promoting humoral IgE responses. Stress surveillance by IELs can thus recognize and remove damaged and possibly (pre-)malignant cells, promote tissue repair, and induce type 2 immunity, which in turn controls inflammation, expels or inactivates noxious substances, and promotes morphological tissue homeostasis.

Immune surveillance can function by many (non-exclusive) mechanisms (Figure 2): (1) recognize and remove damaged, stressed, senescent, and (pre)-malignant cells, (2) remove damaging substances, waste, and dead cells, (3) facilitate re-establishment of homeostasis by repair mechanisms, (4) neutralize potential harmful environmental substances, or (5) dampen detrimental inflammatory reactions.

In relation to cancer immune surveillance, the main focus has been on type 1 immunity and cytotoxic mechanisms, both of which have overwhelming experimental support for playing a role in extrinsic tumor suppression. In both genetic and carcinogen-induced tumor models, cytotoxic molecules such as perforin and TRAIL, as well as NKG2D engagement, have repeatedly been shown to be important in tumor control. Likewise mice lacking type 1 molecules such as IFNγ, IFNGR, IL-12, or type I IFN receptors are significantly more susceptible to carcinogenesis in several models. Mice lacking IFNγ, perforin, TRAIL, functioning FasL or IL-12 responsiveness can also develop spontaneous tumors of variable origin [extensively reviewed in Ref. (139)].

However, the repair functions of immune surveillance are clearly also very important in the early phases of immune surveillance. This is illustrated by the link between wounding and tumor development. It has been observed that tumors can develop at the site of chronic skin wounds or untreated mouth ulcers (140) and there are several case reports of lung metastasis at sites of accidental trauma (141). A clear illustration of the link between a defective wound-healing response and the development of cancer comes from patients with epidermolysis bullosa. These patients have mutations in genes encoding skin extracellular matrix components and suffer from chronic skin blistering and sores – and as a result of the chronic tissue damage are at increased risk of developing squamous cell carcinoma (142, 143). A diminished capacity to repair a damaged barrier can thus predispose to the development of cancer. Such associations between chronic damage/wounds and cancer as well as the histological similarities of wounds and tumors led to the often-cited phrase that “tumors are wounds that do not heal” (144).

The association between chronic wounds and development of cancer may of course not only pertain to the lack of repair *per se* but also to the onset of a detrimental chronic inflammation as a consequence. There is a close association between chronic inflammation and cancer, and once a malignant cell has escaped the early phase of immune surveillance, inflammation can exert prominent pro-carcinogenic effects (145). The tumor-promoting effects of inflammation are being intensively studied and are starting to have implications for the treatment of cancers (145, 146). An important feature of early tumor immune surveillance could thus be the release of anti-inflammatory products in the tissue. Stressed ECs promptly release many anti-inflammatory products such as IL-33, TSLP, and IL-25 – all with the propensity to drive anti-inflammatory type 2 immune responses. The role of such anti-inflammatory DAMPs and type 2 immunity in early cancer immune surveillance remains to be clarified but intriguingly when tumor-protective skin-resident IELs are activated by stressed ECs they promptly release high levels of IL-13 (53) (and Strid-J unpublished data). Interestingly, this IL-13 and a following production of IgE is dependent on engagement of NKG2D on the IELs (53), perhaps suggesting that the tumor-suppressive effect of NKG2D and skin-resident IELs may not solely be via cytotoxic/type 1-mediated immune surveillance mechanisms. The surprising association between NKG2D and anti-inflammatory type 2 immune responses (and IgE) was recently corroborated in a model of allergic pulmonary inflammation, where mice lacking NKG2D were resistant to the induction of allergic inflammation and showed reduced Th2 and IgE responses (147). The association between a stress-sensor such as NKG2D, which has been intimately linked to anti-tumor responses, and induction of type 2 immunity demands a closer look at the role of early type 2 immunity in cancer immune surveillance. The possible role of such early type 2 responses in tissue homeostasis and immune surveillance of cancer as well as its possible pro- and anti-tumor growth functions are discussed in more detail below.

## Role of Type 2 Immunity in Tissue Homeostasis and Immune Surveillance

What is known so far of the physiological role of type 2 responses is that their host-protection properties converge in different forms of barrier defenses (58). This seems logical as epithelial surfaces have a propensity to drive type 2 immunity (rather than type 1) upon non-invasive/non-penetrating challenge or stress and type 2 immune mediators are thus well poised to play a role in early immune surveillance as well as homeostatic tissue regulation. IL-13 is the best-characterized inducer of mucus production and goblet cell hyperplasia in the respiratory and intestinal mucosa. In the skin, transgenic over-expression of IL-13 induces skin remodeling, which is primarily driven by TSLP (148). In both circumstances, hyperplasia results in improved resistance to damage and damaging substances at the body barrier either via production of mucus at the mucosal surfaces or thickening of the skin. IL-13 may also be involved in homeostatic EC differentiation/proliferation in the skin. Epidermal IELs, which are non-redundant for normal tissue homeostasis and wound-healing, are rapid and potent producers of IL-13 following skin challenge (UV-radiation, tape-stripping, NKG2D-ligand expression, and exposure to carcinogen) and mice deficient in IL-13 have delayed barrier repair following epidermal tape-stripping as measured by TEWL (Strid-J unpublished).

Removal or expulsion is another host defense strategy induced by type 2 immunity, which can directly protect against noxious toxins or parasites, as well as limiting their systemic dissemination. The removal/expulsive actions of allergic and type 2 immunity through sneezing/coughing/itching/vomiting/diarrhea are partly induced by EC-derived mediators including TSLP, which acts directly on sensory neurons in the skin triggering itching (149), and by the effect of mast cell-derived histamine on smooth muscles. Type 2 immune mediators can also confer host-protection by inactivation, neutralization, and destruction of noxious substances. This is most notably shown by the requirement for mast cells in the detoxification of snake and bee venom (150) and the evidence that mast cell proteases can specifically attack snake venom at the structures required for toxicity and thereby neutralizing it (151). Recent data strongly suggest that IgE mediates or at least contributes to protection against venoms as for example the protective responses against re-challenge with high doses of bee venom is abrogated in mice lacking B cells, FcεRI, or IgE (56, 57). It is likely that this protection is partly via the very rapid IgE-mediated degranulation of mast cells. Encapsulation and restriction is another layer of barrier defense regulated by type 2 mediators, which can help prevent the spread of noxious substances if elimination or expulsion has been insufficient. Endothelial leakage and exudate formation can be induced by mast cell-derived products and such local tissue edema may impede parasite invasion. For example it has been shown that the edema caused by IgE-mediated mast cell degranulation is important in the defense against macroparasites such as ticks (152). Another restriction mechanism, which may restrict the spread of noxious substance as well as macroparasites, involves sequestration through granuloma formation. Type 2 immune responses protect the host during infection with schistosomiasis by inducing granulomas that sequester the tissue-damaging toxins from the parasite eggs (153).

Perhaps most importantly, much of type 2 immunity seems dedicated to tissue repair and promoting tolerance to damage. Indeed, it has been hypothesized that type 2 immunity has evolved to direct innate wound repair mechanisms (154). The rationale for the induction of tissue repair as a part of type 2 immune defense is obvious. It may also explain the extreme urgency of some type 2 responses (which is not easily explained if directed only toward a slow replicating macroparasite), as damage control may well be more important than pathogen control. In evolutionary terms, it makes sense to be able to quickly expel or neutralize noxious substances as well as rapidly repair the life-essential body barrier. EC-derived cytokine alarmins and cell-ligands can activate and direct the resident tissue cells to promote repair responses; IELs rapidly sense stress and can produce growth factors locally in the absence of further inflammation and tissue-resident ILCs can amplify the type 2 response and produce amphiregulin. Indeed, depletion of ILC2 compromises lung epithelial barrier integrity (155) just as depletion of γδ IEL compromises skin and gut epithelial integrity during homeostasis as well as delaying wound healing (127, 133, 134). Almost all of the cells associated with type 2 immunity are also associated with the wound-healing response. Alternatively activated macrophages produce vascular endothelial growth factor (VEGF), arginase 1, and IGF-1; Eosinophils store preformed growth factors, matrix metalloproteinases, and lipid mediators, all of which can mediate wound healing (156). Amphiregulin is produced by mast cells following FcεRI signaling, potentially also linking IgE responses to wound repair (157). In sum, the effects of type 2 immunity at our body surface tissues play important roles in eliminating, restricting, and neutralizing noxious environmental substances as well as repairing the damage caused and minimizing inflammation – as such this type of immunity is critical for tissue homeostasis and responses to challenges that have breached the epithelial barrier.

**Figure 3:**
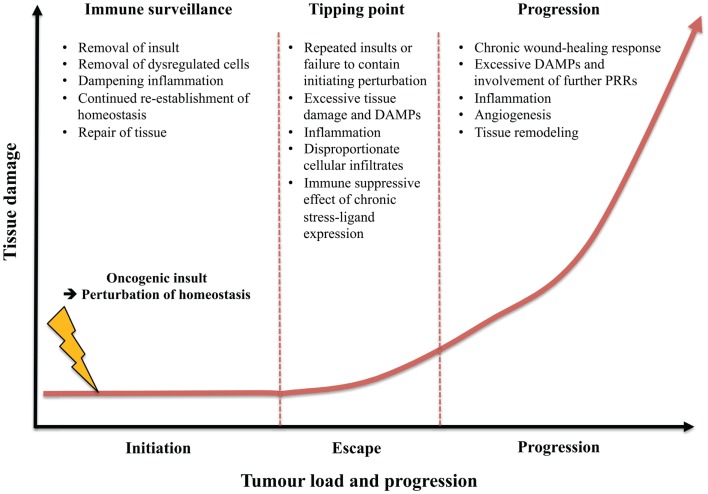
**Contrasting role of type 2 immunity in immune surveillance of early tissue dysregulation versus tumor progression**. The type 2 component of immune surveillance may aid in host-protection against carcinogenesis at epithelial surfaces by removing the oncogenic insult, eliminating the dysregulated cells, dampening excessive inflammation, repairing tissue, re-establishing homeostasis, as well as improving resistance to future damage. However, following continuous perturbations, failure to eliminate the initial insult or dysregulated immune surveillance a tipping point may be reached where excessive tissue damage and DAMPs lead to inflammation, disproportionate cellular infiltrates, and escape from immune surveillance and tissue homeostasis. Once a certain level of tissue damage is reached, a perpetual type 2 response may be detrimental to the host by transitioning to aid carcinogenesis by promoting a chronic wound-healing response and fibrosis as well as supporting neo-angiogenesis.

In terms of early cancer immune surveillance, the role of type 2 immunity has been little explored. However, the effects of rapid type 2 immune responses as outlined above could indeed play a prominent role in cancer immune surveillance. It has been demonstrated that the same tissue-resident IELs act as key components of tumor resistance and potent inducers of type 2 immunity and IgE antibodies (53, 114). The humoral component of this lymphoid stress-surveillance response may limit tissue damage by targeting noxious foreign substances, such as toxins that may be the root cause of the tissue dysregulation. The IgE effector response may promote toxin expulsion and limit their systemic dissemination. Simultaneously, the cellular response can direct cytotoxicity toward dysregulated cells as well as promoting repair of the damaged tissue and dampening inflammation (Figures 2 and 3). To limit the likelihood of cancer, it is clearly important to repair a wound or breached barrier quickly and efficiently as is demonstrated by the close association between chronic tissue damage and cancer. Less efficient wound repair may lead to inefficient immune surveillance against (pre-)malignant cells with damaged cells being allowed to stay longer in the tissue before being replaced. Additionally, slow repair of tissue damage may lead to inflammation, which as discussed can have potent pro-carcinogenic effects.

Although one can imagine a role for type 2 immunity and its regenerative capacity in early cancer immune surveillance this may indeed be a double-edged sword in further development of cancer (Figure 3). Failure of the type 2 response to adequately contain or eliminate the initiating substance may lead to a chronic wound-healing response and exacerbation of inflammation. Such continued tissue damage, repair, and regeneration may ultimately result in fibrosis. Fibrotic tissue is a highly permissive environment for tumor formation and it is also well established that continuous wound-healing responses and tumorigenesis are two processes that rely on similar molecular mechanisms (158). As such it is perhaps not surprising that the literature on type 2/IgE responses and cancer is somewhat bewildering.

## Role of Type 2 Immunity and IgE in Cancer

Both positive and negative effects of type 2 immunity on tumor growth and carcinogenesis have been reported in the literature (Table 1). Contrasting results are clearly in part due to the differing experimental approaches and models but most likely also reflects the divergent roles that type 2 immunity may play at different stages of carcinogenesis and in different tissues. CD4^+^ T cell-derived IL-4 has been reported to induce granulocyte infiltration, thereby promoting tumor clearance (an action enhanced by IL-13), and conversely increase tumor cells’ resistance to apoptosis by up-regulation of anti-apoptotic proteins (159). Many studies carried out in the 1990s and early 2000s demonstrated that tumors or tumor cell lines engineered to produce IL-4 would exhibit increased rejection and retarded growth *in vivo*; curiously a phenotype often dependent on CD8^+^ cytotoxic T cells (160–163). IL-10 has been reported to be both “pro-tumorigenic” by inhibiting tumor cell lysis by cytotoxic T cells and “anti-tumorigenic” by promoting NK-cell-mediated tumor clearance and inhibiting angiogenesis (164). The EC-derived cytokines IL-33 and TSLP have been shown to enhance tumorigenesis by promoting epithelial-mesenchycmal transition (EMT) in organotypic culture of *ex vivo* carcinoma-associated fibroblasts (CAFs) and squamous cell carcinoma cells (165) and by enhancing Th2 inflammation (166). On the contrary TSLP has convincingly been shown in mouse *in vivo* models to be critically important for resistance to skin carcinogenesis (35, 36) establishing TSLP as a tumor suppressor in the skin. Many epithelial cancers express receptors for type 2 mediators such as IL-4 and IL-13, allowing for a direct effect on tumor growth, death, and proliferation that is independent of their effect on immunocytes (167). This perhaps also explains the likely divergent effects of type 2 immunity in early cancer immune surveillance versus in established tumors. Human breast and renal cancer cell lines treated with exogenous IL-13 *in vitro* demonstrate reduced proliferation (168–170) – although an ovarian cancer line demonstrated enhanced invasive and enzyme (protease) activity (171) suggesting IL-13 aids primary tumor invasion and metastasis in this model. No doubt the heterogeneity of cancer cell lines and *ex vivo* tumors derived from patients and experimental animals clouds a consistent description of how potent type 2 cytokines may affect growth and immune surveillance of tumors *in vivo*. Cellular type 2 players, such as eosinophils, have an extensive description in the cancer literature, and have been shown to play a protective role against chemically induced tumors *in vivo* – and directly kill chemically induced fibrosarcomas *in vitro* (172), suggesting efficacious tissue immune surveillance. Mast cells frequently infiltrate the tumor microenvironment and are usually correlated with a poor prognosis in human cancers (173), although both tumor rejection and promotion has been attributed to them in the mouse. Interestingly, the ancient and highly conserved (174) immunoglobulin isotype IgE has been shown to play a significant role in immune surveillance of tumors. Since the 1990s IgE mAbs have been considered for cancer immunotherapy; particularly given IgE’s extreme biological potency and presence of high and low affinity receptors on various effector cell types (175). Indeed, animals deficient in IgE show drastically altered susceptibility to cutaneous chemical carcinogenesis, and an altered tumor cytokine microenvironment (Dalessandri-T and Strid-J; unpublished). IgE-coated irradiated tumor cells has also been shown to generate protective, eosinophil, and T cell immunity to subsequently administered non-irradiated tumors (176). In addition, the type 2 immunoglobulin IgG1 has been reported to be potently tumoricidal when not “blocked” by competing IgG4 antibodies (177).

**Table 1:** **Examples of type 2 cytokines and immunoglobulins influencing tumor pathology**.

Type 2 mediator	Experimental approach	Model	Tumor growth
IL-4	Tumors engineered to produce IL-4, IL-4 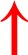	Primary murine renal cancer Injection of syngeneic tumor cell lines	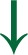 Enhanced CD8^+^ T cell-dependent rejection (160)
			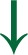 Enhanced CD8^+^ T cell-dependent rejection (161)
			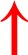 Delayed primary tumor clearance, increased secondary tumor development (178)
			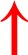 Reduced CD8^+^ T cell-mediated clearance (178)
		Primary murine adenocarcinoma	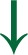 Enhanced CD8^+^ T cell-dependent and eosinophil-mediated rejection (162)
		Vaccination with irradiated tumor cells	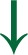 Enhanced CD8^+^ T cell-dependent clearance of lung metastases (163)
	Exogenous rIL-4 treatment, IL-4 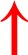	Prostate, breast and bladder cancer cell lines	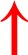 Enhanced resistance to apoptosis and chemotherapeutic agents (159)
IL-13	Tumors engineered to produce IL-13, IL-13 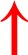	Injection of P815 mastocytoma cell line	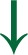 Improved rejection and development of systemic anti-tumor immunity (179)
	Exogenous IL-13 treatment, IL-13 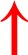	*Ex vivo* leukemic B blasts	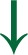 Reduced proliferation and cell-cycle progression assessed by DNA content (168)
		Human breast cancer cell line	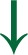 Inhibition of estrogen-induced cell proliferation, unchanged basal proliferation (169)
		Human renal carcinoma cell line	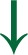 Reduced proliferation and colony formation (170)
		Ovarian cancer cell line	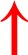 Increased MMP and AP-1-dependant invasion and protease activity in matrigel invasion assay (171)
	Antibody-mediated IL-13 neutralization, IL-13 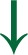	Hodgkin lymphoma cell line	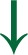 Decreased proliferation and STAT6 phosphorylation (180)
IL-33	IL-33 receptor knockout (ST2^−/−^), IL-33-signaling 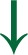	ST2^−/−^mammary carcinoma-bearing mice	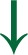 Attenuated tumor growth and metastasis, increased number and cytotoxic activity of NK cells (181)
	Exogenous IL-33 treatment, IL-33 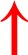	4T1 cell line tumor-bearing mice	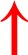 Reduced intra-tumoral tumoricidal NK cells, increased splenic MDSCs and M2 macrophages (182)
	IL-33 co-admin, with HPV DNA vaccine, IL-33 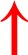	TC-1 cell line (HPV-16 E7-positive) tumor-bearing mice	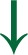 Improved HPV antigen-specific CD4 and CD8 T cells, increased TC-1 regression (183)
	Organotypic culture, IL-33 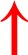	*Ex vivo* human carcinoma-associated fibroblasts (CAFs)	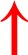 CAFs promote carcinoma invasion via IL-33 signaling and EMT induction (165)
TSLP	Antibody-mediated TSLP neutralization, TSLP 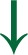	Murine breast tumor xenograft	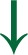 Inhibition of tumor development (166)
	K14-TSLP^Tg^or calcipotriol induced TSLP, TSLP 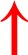	DMBA/TPA chemical skin carcinogenesis	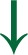 Delayed tumor onset and significantly reduced tumor number and growth (35)
	TSLP receptor knockout or TSLP neutralization, TSLP-signaling 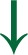	Notchl/Notch2 receptor knockout	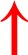 Loss of TSLP-signaling in Notch-deficient epidermis leads to tumor formation (36)
IgE	IgE-loaded tumor cell vaccine, IgE 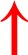	Post-vaccination challenge with RMA lymphoma or MC38 adenocarcinoma	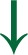 Improved protective eosinophil, CD4^+^ and CD8^+^ T cell responses to tumor challenge (176, 184)
IgGl	Engineered tumor-antigen-specific IgG4, IgGl 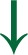	Human melanoma xenograft model	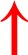 IgG4 blocked potent IgGl-mediated anti-tumor effector functions (177)

## Epidemiological and Clinical Associations between Type 2 Immunity, IgE and Cancer

Associations between allergy history and cancer risk have been investigated in numerous epidemiological studies and their association is being defined in the nascent field of “AllergoOncology” (Table 2). Recent overviews of the epidemiological literature demonstrate that both potent inverse and positive associations exist, which point to complex underlying interactions as well as reflecting the heterogeneity of these diseases. Accordingly, although the relationship between cancer and allergy has intrigued researchers for decades, the biological nature of this association remains unclear.

**Table 2:** **Proposed hypotheses explaining associations between type 2 immunity/allergy and cancer**.

Hypothesis	Predicted allergy– cancer relationship	Predicted affected tissue site		Proposed mechanisms
Antigenic stimulation or chronic inflammation (185)	Positive, causal	All sites	∙	Allergic inflammation and oxidative damage promote pro-tumorigenic gene mutations
			∙	Type 2-induced tissue remodeling and angiogenesis promotes tumor growth and invasion
Inappropriate Th2 skewing (186)	Positive, causal	All sites	∙	Diversion away from protective cytolytic type 1 responses
			∙	Non-protective IgE clonality, or poorly tumoricidal IgG4 class switching with immunosuppressive IL-10
Immune surveillance (93)	Inverse, causal	All sites	∙	Potent effector cells, including γδT cells, mast cells and eosinophils eradicate tumors
			∙	Tumor-specific IgE potently cytolytic via ADCC
			∙	Type 2 immunity repairs tissue damage and dampens inflammation hereby restricting tumor formation
Prophylaxis (55)	Inverse, causal	Mucosal and external surfaces	∙	Tissue type 2 immunity removes or neutralizes noxious and potentially carcinogenic environmental moieties before they cause genotoxicity
			∙	Type 2 immunity restricts systemic dissemination of noxious substances and enhances natural barrier defenses

Two hypotheses put forward to explain positive allergy–cancer associations are the “antigenic stimulation”/“chronic inflammation” hypothesis and the “inappropriate Th2 skewing” hypothesis (Table 2). The “antigenic stimulation” hypothesis was first proposed in the late 1980s (185) and has been reiterated numerous times, also termed the “chronic inflammation” hypothesis (186). This hypothesis proposes that inflammation associated with allergic disease establishes a tissue environment conducive to tumor growth. Indeed, more than 100 years ago a link between inflammation and cancer was first proposed by Virchow (146), who noted the presence of leukocytes in neoplastic tissues and suggested cancer originated at sites of chronic inflammation. Tissue damage with the release of DAMPs, chronic infection, and inflammation are all believed to contribute to the development of malignant disease. Mechanistically, cellular Th2-mediators such as macrophages promote oxidative damage through production of iNOS and hydrogen peroxide via the respiratory burst, increasing the likelihood of damage and mutation of tumor-suppressor genes or cell-cycle regulator genes. Tissue remodeling and pro-angiogenesis factors such as vasoactive mediators from tissue-resident mast cells and eosinophils, as well as VEGF, arginase and matrix metalloproteases released by macrophages, may promote local invasion of outgrowing tumors, and eventual metastasis with establishment of distal secondary loci worsening clinical outcome. Thus, the “antigenic stimulation/chronic inflammation” hypothesis predicts a positive relationship between allergic disease and cancer in any tissue site and this relationship is directly causal, i.e., inflammation secondary to or as a result of allergic disease directly promotes oncogenesis. The “inappropriate Th2 skewing” hypothesis (186) suggests that type 2 mediators – such as IL-4, IL-10, IL-13 – may redirect tissue immunity away from a potently anti-tumor and cytolytic Th1 response, toward an ineffective Th2 response, where IgE is produced and directed toward allergens and not tumor-specific or tumor-associated antigens. Additionally, with production of immunomodulatory IL-10, Th2-immunogloublin IgG4 class-switch recombination is favored over IgE, the former being far less potently tumoricidal, further attenuating anti-tumor responses. This hypothesis therefore also predicts a positive relationship between allergic disease and cancer, in any tissue site, and this relationship is directly causal – skewing to type 2 responses that are non-protective and aids oncogenesis.

Two hypotheses put forward to explain inverse allergy–cancer associations are the “immune surveillance” hypothesis, and the “prophylaxis” hypothesis (Table 2). Prophylaxis was first proposed by Profet (55), and suggests that the symptoms and mechanisms of allergic disease serve to repel and clear potentially mutagenic substances at the external body surfaces before mutagenesis can occur; a coopted function of type 2 immunity which also serves to expel parasites and helminths. Itch induced by type 2 mediators such as TSLP, goblet cell hypersecretion of mucus, sneezing, coughing, vomiting, and diarrhea all act as repulsive mechanisms and are particularly common allergy symptoms. In additional to physical expulsion, type 2 cellular players directly deactivate noxious xenobiotics. In mice at least, mast cells have been shown to degrade venom components through release of carboxypeptidases (150), and IgE raised to a conserved component (and allergen) of many venoms is protective against a repeat exposure (56, 57). The unpleasantness of allergy symptoms also conditions the animal to avoid potentially carcinogenic triggers. Thus, the “prophylaxis” hypothesis predicts an inverse, causal relationship between allergic disease and cancer, particularly at the exposed body barrier surfaces. This hypothesis requires that moieties encountered at the body surfaces are directly carcinogenic, or are pro-carcinogens, and predicts that individuals with allergy symptoms should present with (i) lower levels of carcinogens in their blood and (ii) restrictive or obstructive disease at mucosal surfaces – or that treatment to reduce allergic symptoms results in greater vulnerability to cancer at those sites (187). These corollaries of the “prophylaxis” hypothesis have been poorly investigated. The “immune surveillance” hypothesis, first proposed by Burnet (93), also predicts an inverse allergy–cancer relationship and inverse associations are predicted at any body site. It suggests that allergy and atopic symptoms are indicative of an immune system that is generally hyper-responsive to challenge, and has enhanced immune surveillance capability. Potently cytolytic type 2 responses raised against tumor-associated or -specific antigens can rapidly eradicate dysregulated and proto-neoplastic cells; hence allergy symptoms are a fortuitous, albeit unpleasant, result of an individual’s potent immune system, which also controls dysregulated cells and results in an inverse allergy–cancer relationship. Although the inverse allergy–cancer relationship proposed by the “immune surveillance” hypothesis was originally thought to be purely correlational, as argued in this review, type 2 immunity is also likely to play an important role in early immune surveillance in a direct casual manner by virtue of its ability to remove noxious substances, repair tissue damage and dampen initial inflammation.

In spite of many speculations and associations there is little evidence for a strong association between allergy and *overall* cancer risk (188). However, given that allergic disease occurs primarily at outer epithelial surfaces it is logical to examine the incidence of cancer at specific tissue sites, particularly those at which allergic disease is prevalent; such as the skin, respiratory, and gastrointestinal tracts. A recent large meta-analysis of more than 400 studies of relationships between allergy and cancer reported a preponderance of inverse allergy–cancer associations, and interestingly this was particularly strong for cancers of tissues that interface with the external environment, such as skin, mouth, throat, colon, and cervix (187). These results support the “prophylaxis” as well as the “immune surveillance” hypotheses. Intriguingly, while most studies investigate the link between specifically Type I allergic disease and cancer, a significant inverse association between contact (Type IV) hypersensitivity and breast and non-melanoma skin cancer has been reported, and the authors suggested these data support the “immune surveillance” hypothesis (189).

The large number of published association studies nevertheless often paints a conflicting picture, some of which may be due to methodological constraints. Most retrospective studies on allergy–cancer associations have investigated self-reported or clinician-diagnosed allergy, methodologies particularly prone to recollection and reporting bias (former) and subject selection bias (latter). Researchers have attempted to alleviate these concerns at least in part by discriminating subjects on the basis of physiological indices of allergic disease, such as serum IgE titers (190, 191) and skin-prick testing. Of course, serum total or allergen-specific IgE suffers less from human biases, but these are not a definite metric of allergic status in all individuals, all of the time; in addition this methodology potentially precludes the possibility of examining significant non-Type I hypersensitivities in the analysis. Another concern is that if variation between individual’s allergy symptoms is due more to differences in individual’s exposure to antigens and/or carcinogens (the environment as a confounding variable), rather than individual differences in immunity (“immune surveillance” hypothesis), then positive correlations between allergic symptoms and cancer may occur – even if the “prophylaxis” or “immune surveillance” hypotheses are true. Particularly, given that exposure to carcinogenic allergens (such as cigarette smoke or vehicle exhaust) results in increased cancer and allergic disease (187). In addition, the genetic and phenotypic heterogeneity of tumors, and the complex inflammatory niche in which they reside, may also confound epidemiological association studies.

## Concluding Remarks

Epithelial cancers are products of a series of events starting with dysregulated and stressed ECs. It is now clear that challenges to ECs can trigger discrete pathways promoting the release of specific cytokines, chemokines, and expression of stress antigens on the EC surface. Together this can powerfully drive immune responses, initially from cells resident in the epithelial and subepithelial compartment. The initial response to EC challenge and damage is often a very rapid type 2 immune response. This may serve to remove or neutralize noxious challenging substances, clear waste, repair the tissue, dampen inflammation, and re-establish tissue homeostasis. It may also directly contribute to the elimination of damaged cells together with cytolytic mechanisms from resident IELs and other immunocytes. Rapid type 2 immune responses at body surfaces thus prominently contribute to immune surveillance of dysregulated ECs. Since epithelial dysregulation contributes notably to a multitude of inflammatory diseases, this may not only be important in control of (pre-)malignancy but could be important at disease-initiating stages in a variety of diseases.

Immune surveillance by its nature is mainly important in the initiation phase of tissue damage and tumor control – for maintenance of tissue homeostasis. The same effector molecules and mechanisms may play a very different role during the progression phase of tissue damage and tumor growth. Failure to eliminate the original damaging substance, damaged cells, or to repair the tissue may lead to a continual stress response with excessive release of DAMPs, persistent stress-ligand expression, inflammation, and a chronic wound-healing response and this may determine the transition point between beneficial and detrimental functions of type 2 immunity in the course of disease. Similarly, continual exposure to noxious environmental substances may eventually overwhelm the immune surveillance mechanisms keeping the damage in check and result in pathology or tumor growth. Further research is needed to study whether the balance between tumor cell growth and elimination may be tipped back upon immune manipulations aimed at enhancing naturally occurring immune surveillance.

## Conflict of Interest Statement

The Guest Associate Editor Fang-Ping Huang declares that, despite being affiliated to the same institution as authors Tim Dalessandri and Jessica Strid, the review process was handled objectively and no conflict of interest exists. The authors declare that the research was conducted in the absence of any commercial or financial relationships that could be construed as a potential conflict of interest.
